# Interest of Ultrasonographic Assessment of Diaphragmatic Function in Cardiac Rehabilitation Center: A Case Report

**DOI:** 10.1097/MD.0000000000000801

**Published:** 2015-05-21

**Authors:** Alain Boussuges, Guillaume Chaumet, Laurent Poirette

**Affiliations:** From the UMRMD2, Aix-Marseille University and IRBA, Marseille (AB, GC); Unité de Réhabilitation Cardiologique, Hôpital Léon-Bérard, Hyères, France (LP).

## Abstract

Diaphragmatic paresis is a rare but recognized complication of atrial fibrillation ablation.

A 59-year-old woman experiencing dyspnea in supine position and for minimal effort was admitted in a cardiac rehabilitation center. One month before, she was referred to a cardiac center to ablation of paroxysmal atrial fibrillation. After the procedure, the patient developed respiratory failure attributed to aspiration pneumonia and requiring mechanical ventilation.

At admission in the rehabilitation center, M-mode ultrasonography reported an absence of movement of the right hemidiaphragm during quiet breathing and a paradoxical movement during voluntary sniffing.

Chest ultrasonography can be useful to detect diaphragmatic dysfunction in patients suffering from dyspnea, at admission in a cardiac rehabilitation center. Its use should be envisaged more frequently.

Diaphragmatic paresis is a rare but recognized complication of atrial fibrillation ablation. In a multicenter study,^1^ this complication has been assessed around 0.48% (from 0.37% to 1.6%, in function of the medical institution). The phrenic nerve is injured during the procedure through the vessels or atria wall. Right phrenic nerve can be damaged during right superior pulmonary vein or superior cava vein disconnection. Furthermore, ablation at the left atrial appendage can injured left phrenic nerve. Depending on ablation procedure, nerve dysfunction can be related to electrical current or thermal lesions.^2^ We report a case of right diaphragmatic paralysis after atrial fibrillation ablation. Respiratory troubles were attributed to pneumonia. Diagnosis was performed using chest ultrasonography upon admission to the rehabilitation center. This case has not been published and supported the usefulness of ultrasonography in rehabilitation center.

## CASE DESCRIPTION

A 59-year-old woman was referred to a cardiac center for ablation of paroxysmal atrial fibrillation. This woman suffered from obesity (height: 170 cm, weight: 112 kg, body mass index: 38.7). Atrial fibrillation ablation was performed under general anesthesia. Orotracheal intubation was difficult and complicated by pulmonary aspiration of gastric content. Subsequently, no incident was recorded during atrial fibrillation ablation. After the procedure, the patient developed respiratory failure, requiring mechanical ventilation. In the intensive care unit, respiratory failure was attributed to a bilateral pneumonia. Antibiotherapy was introduced. Thereafter, respiratory function slowly improved and the weaning could be obtained 16 days after the orotracheal intubation.

The patient was admitted to the cardiac rehabilitation center 30 days after atrial fibrillation ablation. She suffered from dyspnea in supine position and for minimal effort. Electrocardiogram reported a normal sinus rhythm. Echocardiography showed normal left ventricular systolic function (ejection fraction: 57%). Left atrial diameter was enlarged (48 mm). No significant valvular disease was recorded. The ratio of transmitral Doppler early filling velocity to tissue Doppler early diastolic mitral annular velocity (*E*/*E*′ ratio) was calculated as 5.4 suggesting that left ventricular filling pressures were not increased. Pulmonary arterial pressure was estimated around 50 mm Hg. Thoracic X-ray reported right hemidiaphragm elevation. Pulmonary function test demonstrated a restrictive pattern. Forced vital capacity was 1.42 L (46% of predicted value – 3.08 L) and forced expiratory volume in 1 second was 1.1 L (42% of predicted value – 2.62 L). Arterial blood gas analysis reported hypoxemia (*p*O_2_ = 57 mm Hg).

**FIGURE 1:**
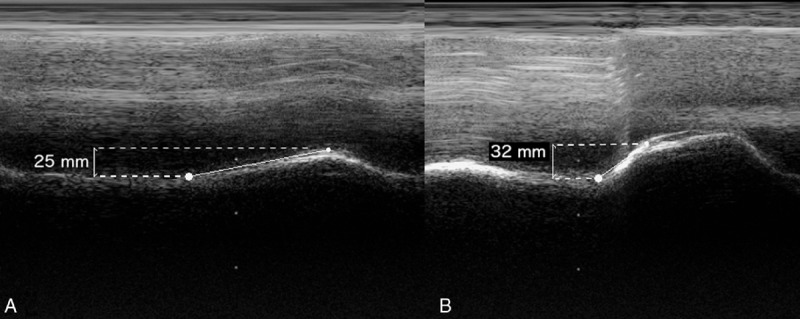
Left hemidiaphragmatic motion recorded in M-mode sonography during (a) quiet breathing and (b) voluntary sniffing.

**FIGURE 2:**
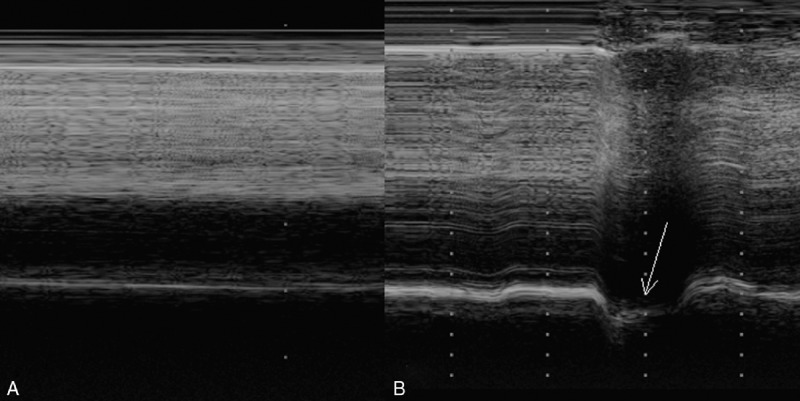
Right hemidiaphragmatic motion recorded in M-mode sonography: absence of movement during (a) quiet breathing and (b) paradoxical movement (arrow) during voluntary sniffing.

Chest ultrasonography was performed using a Philips iE33 ultrasound system (Andover, MA) equipped with a 1 to 5 MHz transducer (S5-1 probe). Left hemidiaphragm mobility was normal during quiet breathing and voluntary sniffing (Figure 1). Furthermore, right hemidiaphragm mobility was nil during quiet breathing (Figure 2a). In addition, a paradoxical movement was recorded during voluntary sniffing (Figure 2b). The patient provided informed consent to be reported in this manuscript.

## DISCUSSION

At admission in a cardiac rehabilitation center, many patients suffer from cardiac and respiratory disease. To adopt an appropriate rehabilitation protocol, it is important to assess both cardiac and respiratory function. In this context, it is interesting to assess lung volume and flow. Furthermore, the study of diaphragmatic function is of interest. Indeed, in cardiac patients, phrenic nerve injury can result from numerous surgical or medical invasive procedures such as extracorporeal circulation, central vein cannulation, pacemaker battery change and ablation for recurrent arrhythmias.^1,3,4^ Clinical troubles induced by diaphragm paralysis can take a wide variety of clinical form.^5^ In some cases, the patients are asymptomatic. However, in subjects with preexisting respiratory disease or in the case of bilateral phrenic nerve injury, the patients can suffer from acute respiratory failure. The more frequently, the dyspnea is slight, and occurs at exercise or in supine position. Respiratory troubles induced by diaphragm paralysis could be also attributed to another disease. Lastly, some intercurrent diseases mask the diaphragmatic dysfunction. In the reported case, the severity of the respiratory troubles has been attributed to aspiration pneumonia, in a patient suffering from pulmonary restrictive syndrome secondary to obesity. In this context, the diagnosis of diaphragm paralysis can be delayed.

The diagnosis of diaphragm paralysis is commonly based on chest radiography. The elevation of 1 or both leaves of the hemidiaphragm is reported in the event of diaphragmatic paralysis. Nevertheless, this radiological anomaly is little specific, indeed hemidiaphragm elevation can be secondary to lung or pleural disease. Other methods such as fluoroscopy, respiratory function tests or phrenic nerve testing have been used. M-mode ultrasonography has demonstrated his interest^6^ and can be advantageously used at admission in a rehabilitation center. Indeed, all cardiac centers have an ultrasound system and the method to assess diaphragmatic function^7^ is easy to learn for a physician. In the patient affected by diaphragmatic paralysis, the hemidiaphragm mobility is null during quiet breathing. Furthermore, voluntary sniffing induces a paradoxical movement.

The diagnosis of diaphragmatic paralysis is important before the beginning of the rehabilitation. Indeed, it has been demonstrated that inspiratory muscle training may improve inspiratory muscle strength and increase paralyzed diaphragm mobility, in cardiac surgery patients suffering from diaphragm paralysis.^8^ The recovery of diaphragmatic function depends on the nature of the damage. Cold lesions seem to induce better prognosis than mechanical lesions. In a multicenter study, on a population of patients suffering from diaphragm paralysis induced by atrial fibrillation ablation,^1^ after a mean follow-up of 3 years, 66% of the population studied had complete recovery, 17% had partial recovery, and 17% had no recovery. The average delay of recovery was 4 months after the injury. Consequently, repeated ultrasonographic examinations are interesting during the rehabilitation period. In some patients, with no recovery and incapacitating respiratory troubles, diaphragmatic plication^9^ can be proposed.

## STUDY LIMITATIONS

The limitation of this case report is that just 1 patient is described. Further studies are needed to assess the contribution of chest ultrasonography in the management of patients in cardiac rehabilitation centers.

## CONCLUSIONS

The interest of ultrasonography in rehabilitation centers has been recently emphasized.^10^ This case report supported the utility of M-mode ultrasonography at admission in a cardiac rehabilitation center to detect diaphragmatic dysfunction. In patients suffering from diaphragm paralysis, serial ultrasonographic evaluation can be proposed to monitor the potential recovery of diaphragm function.
